# Determinants of COVID-19 Vaccine Acceptance and Hesitancy: A Systematic Review

**DOI:** 10.3390/vaccines12121352

**Published:** 2024-11-29

**Authors:** Juliana Soares Tenório De Araújo, Felipe Mendes Delpino, Rubia Laine de Paula Andrade-Gonçalves, Francisca Bruna Arruda Aragão, Letícia Perticarrara Ferezin, Denise Alves Santos, Neemais Costa Duarte Neto, Murilo César do Nascimento, Simão Pedro Tavares Moreira, Gabriela Ferreira Ribeiro, Rayssa Francielly dos Santos Alves, Ricardo Alexandre Arcêncio

**Affiliations:** 1 Campus São Paulo, University of São Paulo, São Paulo 05508-220, SP, Brazil; rubia@eerp.usp.br (R.L.d.P.A.-G.); aragao_bruna@usp.br (F.B.A.A.); niisealvessantos1@gmail.com (D.A.S.); 2 Campus Pelotas, Federal University of Pelotas, Pelotas 96010-610, RS, Brazil; fmdsocial@outlook.com; 3 Ribeirão Preto College of Nursing, Campus Ribeirão Preto, University of Sao Paulo, Ribeirão Preto 14040-902, SP, Brazil; lehferezin@usp.br (L.P.F.); ricardo@eerp.usp.br (R.A.A.); 4 Campus São Luís, Federal University of Maranhão, São Luís 65020-070, MA, Brazil; neemiascosta50@gmail.com; 5 Nursing School, Campus Sede, Federal University of Alfenas, Alfenas 37130-001, MG, Brazil; murilo.nascimento@unifal-mg.edu.br; 6 Campus Maceió, State University of Health Sciences of Alagoas, Maceió 57010-300, AL, Brazil; simaoptm@gmail.com (S.P.T.M.); gabriela.ribeiro@academico.uncisal.edu.br (G.F.R.); rayssa.alves@academico.uncisal.edu.br (R.F.d.S.A.)

**Keywords:** vaccination, vaccine acceptance, vaccine hesitancy, COVID-19

## Abstract

**Background/Objectives:** COVID-19 is an infectious disease whose prevention is significantly aided by vaccination, which reduces both case severity and mortality. Despite the safety and efficacy of vaccines, acceptance is not universal, and understanding of the factors influencing vaccination decisions and hesitancy remains limited. This review aims to identify and analyze studies addressing two key questions: what influences the decision to vaccinate and what factors are associated with vaccine hesitancy. **Methods:** This systematic review was conducted following the PRISMA guidelines. Data collection utilized descriptors related to vaccine adherence and hesitancy, based on the PEO strategy of the Joanna Briggs Institute (JBI). Searches were conducted in Embase, Scopus, PubMed, Lilacs, and Web of Science, focusing on publications from 2021, the year the first COVID-19 vaccine was approved. After excluding duplicates and selecting articles based on eligibility criteria, the analysis involved data extraction and methodological quality assessment using JBI tools. **Results:** A total of 5268 publications were identified, with 30 included in this study. Factors associated with vaccine hesitancy included low education levels, social media influence, confidence in vaccine safety, and fear of side effects. In contrast, factors linked to vaccine acceptance included higher education, higher income, older age, and existing comorbidities. **Conclusions:** The findings highlight the urgent need for targeted health communication and education strategies, particularly for vulnerable groups. Public health policies should incorporate these factors to enhance vaccination adherence and build public confidence in vaccine safety, which is essential for mitigating future health emergencies.

## 1. Introduction

COVID-19 is an infectious disease that can be prevented through vaccination. The vaccine not only protects against infection but also reduces the severity of cases, thereby decreasing the associated morbidity and mortality rates [1,2]. Since the onset of the COVID-19 pandemic, over 6 million people have lost their lives due to the infection, highlighting the seriousness of the global challenge we still face [2].

Despite advances in vaccination, the global immunization rate remains below the target set by the World Health Organization, which aimed to achieve 65.09% of the world population fully vaccinated by 12 August 2024 [3]. This situation poses a significant obstacle to controlling the pandemic and highlights the need for ongoing efforts in awareness campaigns and access to vaccines.

It is well known that vaccination is an effective and safe tool in the fight against COVID-19, and its progress has significantly reduced the occurrence of severe cases and deaths [1,2]. Thus, vaccines are a key element in the package of measures aimed at controlling the spread of the new coronavirus. In this context, despite being a health investment with excellent cost-effectiveness, resulting in a tremendous impact on health by preventing millions of deaths each year, vaccine acceptance is not universal [4].

There are gaps in knowledge regarding the factors associated with individuals’ decisions to get vaccinated against COVID-19, as well as the behavioral patterns related to vaccine hesitancy during the pandemic. This evidence is crucial for health institutions and organizations to prepare for and effectively address future public health emergencies. Through a review, it becomes possible to assess the state of the art concerning the factors that influence an individual’s decision to vaccinate. This information can be valuable for health managers in developing awareness campaigns about the importance of vaccination.

The aim of this review is to identify and analyze studies addressing two key questions: what influences the decision to vaccinate and what factors are associated with vaccine hesitancy.

## 2. Materials and Methods

### 2.1. Type of Study

This is a systematic review conducted according to the Preferred Reporting Items for Systematic Reviews and Meta-Analyses (PRISMA) [5] guidelines and registered with PROSPERO (International prospective register of systematic reviews; Centre for Reviews and Dissemination, University of York, York, UK; https://www.crd.york.ac.uk/prospero/ (accessed on 8 April 2024)) CRD42024534308. The guiding questions were: “What factors are associated with the decision to get vaccinated?” and “What factors are associated with vaccine hesitancy?” We used the PEO (Population, exposure and outcome) strategy proposed by the Joanna Briggs Institute 6 for systematic reviews, which recognizes this approach as the most suitable for formulating questions about the effects of exposure.

### 2.2. Search Strategy

The searches were conducted in April 2024, without language restrictions. We searched for studies published from 2021 to 2024, as that was the year the first COVID-19 vaccine was approved for use. The searches were carried out in the following databases: Excerpta Medica database (Embase^®^—https://www.embase.com), Scopus, owned by Elsevier (https://www.scopus.com), PubMed (https://pubmed.ncbi.nlm.nih.gov/), and the Latin American and Caribbean Health Sciences Literature (LILACS—accessed through the Regional Portal of the Virtual Health Library—https://pesquisa.bvsalud.org/portal/advanced (accessed on 8 April 2024)), as well as Web of Science. The searches were conducted using vocabulary in English.

We conducted the searches using specific strategies for each database, along with the boolean operators AND and OR. The boolean operator OR was used between words within the same group (“word” OR “word”), while AND was used between sets of words from different groups (“set of words from group_1_” AND “set of words from group_2_” AND “set of words from group_3_”).

### 2.3. Inclusion Criteria

Only primary observational and analytical studies that addressed factors associated with vaccination adherence or hesitancy regarding COVID-19 within the general population were included. The selected articles needed to be published from 2021 onward, as COVID-19 vaccines began to be implemented that year.

### 2.4. Exclusion Criteria

Studies that did not address the general population, such as research focused on specific population groups, were excluded from the review, along with articles that did not directly answer the outlined research questions. Additionally, comments, editorials, reviews, case reports, protocols, theses, dissertations, and conference abstracts that did not provide primary or analytical data were also excluded.

### 2.5. Export of Studies

The search results were exported to Rayyan, a free online tool that uses artificial intelligence to facilitate study screening in systematic reviews. After removing duplicates, two independent reviewers screened the titles and abstracts of the remaining studies. Those that met the eligibility criteria were selected for full-text reading, where a new screening was conducted to identify the studies to be included in the review. This rigorous process ensured the inclusion of relevant and methodologically sound studies.

### 2.6. Quality Assessment

The methodological quality of the articles was assessed using specific tools proposed by the Joanna Briggs Institute (JBI) [6]. In this case, tools for evaluating cross-sectional studies were used, allowing us to indicate the number of items adequately addressed in the studies based on the number of items outlined by the tools (eight items for cross-sectional studies). Limitations noted in the articles were also considered to complement this evaluation. No study was excluded based on the methodological quality assessment.

### 2.7. Data Analysis

The included articles were subjected to a narrative synthesis after data extraction, performed by one reviewer and verified by another. For this, a specific instrument based on Ursi was used, which included the following variables: article title, authors, journal name, year of publication, study type, objective, sample size, main results, and methodological quality assessment. The results were presented in summary tables and complemented by the analysis in the discussion.

## 3. Results

### 3.1. Selection of Studies

**Figure 1:**
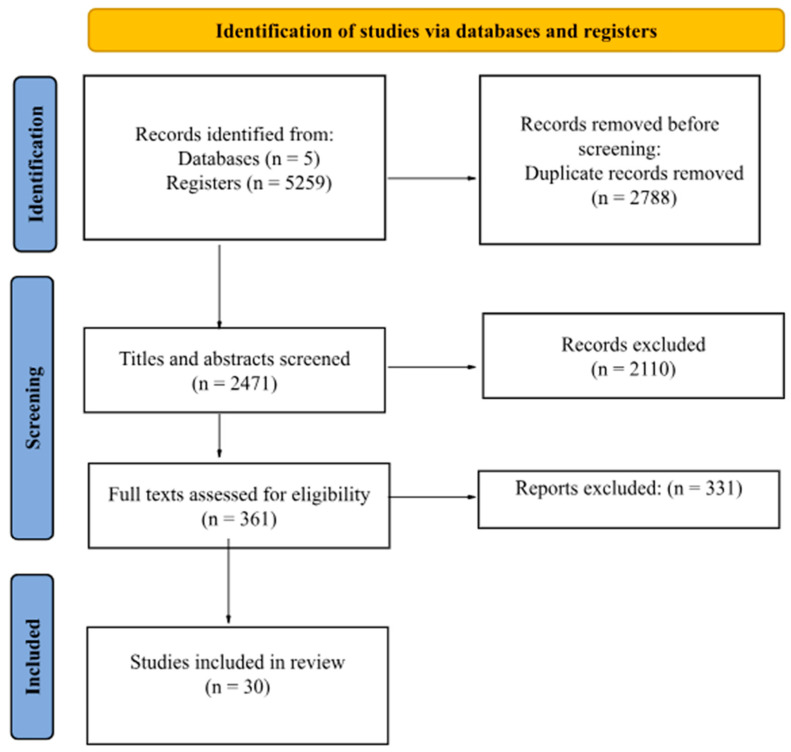
Flow diagram of articles selection stages.

The search identified 5259 scientific publications, of which 2788 were excluded for being duplicates and 2471 were retained after reading their titles and abstracts. As a result, 361 publications were deemed eligible for full-text reading, of which 331 were excluded for not meeting the inclusion criteria, leaving 30 articles in the review (Figure 1).

### 3.2. Characteristics of the Studies

The studies included in the review were conducted across different continents, including Africa, Asia, North America, and Europe, encompassing diverse populations (Supplementary Material Table S1). They were published between 2021 and 2023 and focused on the general population to investigate factors related to COVID-19 vaccination. The articles primarily used observational and analytical methodologies, with sample sizes ranging from hundreds to thousands of participants. Data sources included cross-sectional surveys and questionnaires administered in specific populations, both in urban and rural areas.

These studies analyzed sociodemographic, behavioral, and psychological factors, aiming to identify patterns of vaccine acceptance or hesitancy. Notable methods included structured questionnaires and in-person interviews, with data collected directly from populations or through online platforms. Most studies included variables such as age, education, income, exposure to information, and beliefs related to vaccine safety.

Some studies addressed large populations, such as one conducted in nine countries that included 9264 participants, while another study in Egypt had 24,376 participants. Smaller samples were also common, such as in the study conducted in Saudi Arabia with 531 participants. The majority of the studies included in this review, i.e., 22 studies, had more than 1000 participants. Overall, sample sizes varied from a few hundred to tens of thousands, depending on the country or region of interest. These samples were mainly collected through cross-sectional designs, allowing for the assessment of data at a single point in time.

The characteristics of participants showed significant diversity across the studies, reflecting the sociodemographic variability of the analyzed countries. Most studies had a high participation of women, such as the study conducted in nine countries, where the sample was predominantly females aged 55 years or older. In contrast, other studies, like the one in Ghana, presented a more balanced profile between men and women, with an average age of 40 years. Educational levels varied significantly among the studies, with some highlighting a high percentage of participants with higher education, while others, such as those in Ghana and Nigeria, included a significant proportion of individuals without formal education or with lower educational levels. Additionally, factors such as marital status, occupation, and religious beliefs influenced vaccine hesitancy in various regions, demonstrating the complexity of sociocultural predictors in vaccine acceptance.

### 3.3. Main Results

The main findings from the studies included in the systematic review reveal a diverse landscape of factors associated with COVID-19 vaccine acceptance and hesitancy across different populations and regions of the world. As described in Table 1 and Table 2, in many studies, vaccine acceptance was strongly linked to sociodemographic characteristics such as higher education levels, greater income, older age, and the presence of comorbidities. Individuals with higher educational attainment were more likely to accept vaccination, partly due to a better understanding of the vaccine’s benefits and greater trust in available scientific information.

Conversely, vaccine hesitancy was often associated with fear of side effects, distrust regarding the efficacy and safety of vaccines, and reliance on information from unreliable sources, such as social media. In several countries, social media use was identified as a significant negative predictor for vaccine acceptance, particularly in regions where misinformation about vaccination effects was widely disseminated. Additionally, psychological factors, such as high levels of stress and distrust in government or health authorities, also contributed to hesitancy.

The studies highlighted that younger populations and those with lower education levels showed greater resistance to vaccination. Fear of long-term adverse effects, combined with the belief that the vaccine was developed too quickly, significantly contributed to this hesitancy, especially in contexts where information about the vaccine development process was not adequately disseminated. In some regions, religion and cultural beliefs also played an important role in the decision to get vaccinated or not.

Another relevant finding was the association between trust in health institutions and vaccine adherence. Studies conducted in contexts where vaccination campaigns were widely supported by trusted authorities, such as doctors and community leaders, demonstrated higher vaccine acceptance. In contrast, in countries or regions where the population had less confidence in government authorities or the healthcare system, vaccine hesitancy was more prevalent.

### 3.4. Quality of the Studies

The assessment of the methodological quality of the studies included in the review was conducted using specific tools proposed by the Joanna Briggs Institute (JBI), applicable to cross-sectional studies. Each study was evaluated based on eight criteria related to methodology, such as sample adequacy, clarity of objectives, and rigor in data analysis. Scores ranged from 5 to 8 points, with 8 being the maximum possible score. The average score across the studies was 6.4, indicating that most met the methodological criteria satisfactorily, despite some identified limitations.

The studies with lower scores exhibited limitations primarily related to sample size and representativeness, as well as potential selection biases. In contrast, the studies with higher scores demonstrated consistent methodological rigor, featuring representative samples and robust analyses that clearly addressed the factors associated with vaccine acceptance and hesitancy. No studies were excluded based on methodological quality; however, the identified limitations were taken into account in the interpretation of the results.

## 4. Discussion

The objective of this systematic review was to evaluate the factors associated with COVID-19 vaccine acceptance and hesitancy. The analysis of the 30 included studies revealed a multifactorial landscape influencing the population’s decision to engage in vaccination. Key findings highlighted sociodemographic factors, such as education level, income, age, and the presence of comorbidities, which were related to higher vaccine acceptance. Conversely, fear of adverse effects, distrust in vaccine safety, and exposure to misinformation—particularly through social media—were the factors most associated with vaccine hesitancy.

The interpretation of these findings suggests that vaccine acceptance is closely tied to access to information and the level of trust in health authorities. Individuals with higher education and income tend to have a clearer understanding of vaccine safety and efficacy, which promotes greater adherence. In contrast, vaccine hesitancy is heavily influenced by the spread of misinformation and fear of side effects—issues that need to be addressed through appropriate communication campaigns. Moreover, trust in government and health institutions plays a crucial role: contexts with high levels of trust show greater vaccine uptake, while distrust exacerbates hesitancy. Given this landscape of various factors that can influence vaccine acceptance, it is essential for health authorities to encourage the adoption of differentiated approaches in their strategies, fostering ongoing dialogue with all segments of the population [37].

A central aspect contributing to vaccine hesitancy, identified in several studies included in this review, is the fear of side effects and distrust regarding the safety of COVID-19 vaccines. This fear is often fueled by the spread of misinformation, particularly through social media, where rumors and conspiracy theories circulate. In some contexts, this misinformation has been exacerbated by a lack of effective health education campaigns that directly address these fears.

Combating vaccine hesitancy, therefore, requires a coordinated public communication strategy aimed at restoring trust in vaccines by disseminating clear and accessible scientific information, as well as using reliable sources for sharing this information. In Brazil, during the pandemic, this scenario was evident and was linked to information propagated by the government at the time, which contributed to some of the refusals related to vaccination.

This review reveals that sociodemographic factors, such as education level, income, and age, directly influence decisions regarding vaccine acceptance. Individuals with higher levels of education, for instance, tend to be more receptive to vaccination due to a better understanding of the vaccine’s benefits. Conversely, those with lower education levels or limited access to quality information are more likely to exhibit vaccine hesitancy. Inequality in access to information and healthcare services also emerges as a critical issue, especially in less developed or rural areas. To overcome these barriers, it is essential for vaccination campaigns to be directed inclusively, using language and methods that reach these vulnerable groups, ensuring that information is conveyed clearly and understandably.

The results also suggest that trust in health institutions and the healthcare system of each country plays a crucial role in vaccine adherence. In countries where vaccination campaigns were widely supported by community leaders, doctors, and other trusted figures, there was a higher uptake of vaccines. Conversely, in contexts where there is a history of distrust in health authorities—whether due to political reasons or previous management of health crises—vaccine hesitancy was more pronounced. Creating regional campaigns that involve local and community leaders can be an effective strategy to overcome this barrier, ensuring that the message about the importance of vaccination is disseminated in a more trustworthy and accessible manner for the entire population.

The COVID-19 vaccine is one of the key tools for controlling the pandemic, but its success also depends on combating misinformation. The spread of fake news during the pandemic may have contributed to some of these findings. Efforts to counter fake news during the pandemic have helped to improve misinformation to some extent, but this is an action that needs to be ongoing [38,39]. Groups with lower education levels and limited access to reliable information are especially vulnerable to incorrect information. Therefore, evidence-based educational campaigns focused on debunking myths and promoting trust in health authorities are essential. Clear and accessible communication, combined with the dissemination of accurate information, is vital for overcoming vaccine hesitancy and preparing society to face future health crises.

In terms of limitations, some of the included studies presented methodological issues, such as small or poorly representative samples of certain populations, which may have affected the generalizability of the results. Selection biases were also identified in some studies, potentially limiting the accuracy of the conclusions. Additionally, due to the heterogeneity of the studies, it was not possible to conduct a meta-analysis to evaluate the results in an aggregated manner. It is recommended that longitudinal studies be conducted to better understand the risk factors for vaccine adherence or hesitancy, which could contribute to making future vaccination campaigns as successful as possible.

Among the strengths of this review is the geographic diversity of the included studies, spanning different continents and populations, as well as the number of studies incorporated. This diversity provides a comprehensive view of the factors influencing vaccine hesitancy and acceptance in various cultural and social contexts. Additionally, the rigor in the selection and evaluation of studies, following the PRISMA guidelines, ensures that all relevant studies are included and that the best available information in the literature is obtained.

Future perspectives include the need for more longitudinal studies that explore the evolution of factors associated with vaccination over time, particularly in future vaccination campaigns against other diseases. Additionally, evidence-based communication strategies should be prioritized to combat misinformation and increase confidence in vaccination. Awareness campaigns targeted at groups more vulnerable to hesitancy, such as young people and those with lower education levels, can be crucial for increasing vaccination rates and effectively combating future pandemics.

## 5. Conclusions

This systematic review highlighted the key factors associated with vaccine acceptance and hesitancy regarding COVID-19 in different population contexts. Sociodemographic factors, such as higher education, elevated income, and older age, were significant predictors of vaccine acceptance, while fear of side effects, distrust in vaccine safety, and exposure to misinformation were the main factors related to hesitancy. Trust in health institutions also played an essential role, with greater vaccine acceptance in contexts where this trust was higher. These findings are crucial for guiding public policies and awareness campaigns aimed at increasing vaccine adherence.

In light of the challenges posed by vaccine hesitancy, it is essential to invest in more effective communication strategies that clarify doubts and mitigate the impact of misinformation. Additionally, understanding the sociocultural barriers that influence vaccine acceptance can aid in developing targeted interventions for more vulnerable groups. Recognizing these factors is crucial not only for combating the COVID-19 pandemic but also for equipping health authorities to address future public health emergencies more efficiently.

## Figures and Tables

**Table 1:** Factors associated with vaccination adherence against COVID-19.

Category	Associated Factors
Sociodemographic factors	- Age between 18 and 35 [7,8] - High school or less [7,8,9] - Being male [7,8,9,10,11,12] - Older people, being elderly [9,10,11,13,14] - Being self-employed [13] - White skin color [10]
Beliefs experiences during the pandemic	- Protection for themselves and others [15] - Fear of illness [16,17] - Co-morbidities [8,9,18,19]
Sources of information	- Knowledge [11,20,21,22]
Behavioral factors	- Anxiety [16] - Right-wing party [10] - Previous flu vaccination [11] - Perceived risk [12]
Immunobiologicals	- Knowledge about vaccines [9,11,20,21] - Confidence in vaccines [9,11,21]

**Table 2:** Factors associated with vaccine hesitancy against COVID-19, Ribeirão Preto, 2024.

Category	Associated Facctors
Sociodemographic factors	- Female [11,12,16,20,23,24,25,26,27,28]; - Level of education [11,16,20,24,26,27,29] - Age between 34–49, over 45 [20,29]; - Urban dwellers [20,24] - Young people, people on low incomes and people with lower levels of education [11,12,14,16,27] - Married [20] - Employed [20] - Black [11,27]
Experiences during the pandemic	- Stress [30]; - The use of social medial [23] - Psychological distress [12]
Sources of information	- The use of social media and the internet to obtain information [24,30]. - Lack of information [7,23,24,31] - Use of television and Facebook [25]
Immunobiologicals	- Distrust of the vaccine [9,17,23,24]; - Concern about side effects/adverse events [8,12,23,31,32]; - Confidence in the safety and efficacy of vaccines [7,8,9,13,14,20,22,24,33,34,35,36] - Possible long-term health risks associated with vaccines [7,31] - Fear of needles [32] - Belief in acquired immunity [32]
Religion	- Have some religion [13,23,24,28,32],
Behavioral factors	- Racial discrimination [33,34]; - Use of alcohol and tobacco [32]; - Not trusting the government [13,16] - Voters from opposition political parties [24,27]. - Low perception of severity and/or risk. [9,26,31,34,35,36]

## Supplementary Materials

The following supporting information can be downloaded at: https://www.mdpi.com/article/10.3390/vaccines12121352/s1, Table S1. Description of articles included in the systematic review on factors associated with vaccine adherence or hesitancy against COVID-19, Ribeirão Preto, 2024; Table S2. Preparation of the study question according to the PEO strategy, Ribeirão Preto, 2024; Table S3. Bibliographic search strategy used in the systematic review on the factors associated with vaccine adherence or hesitancy against COVID-19, according to the databases researched, Ribeirão Preto, 2024
