# Supplementary material for: Determinants of COVID-19 Vaccine Acceptance and Hesitancy: A Systematic Review
Source: Vaccines (Basel). 2024 Nov 29;12(12):1352. doi: 10.3390/vaccines12121352 (PMC11680215; doi:10.3390/vaccines12121352)
Supplement: Supplementary file 1 [file vaccines-12-01352-s001.zip › vaccines-3318880-supplementay.pdf]

Supplementary Material

Supplementary Material Table S1. Description of articles included in the systematic review on factors associated with vaccine adherence or hesitancy against COVID-19, Ribeirão Preto, 2024.

Title	Authors / Journal / Year of publication	Objective	Sample size and country of study	Study design and sample characteristics	Main findings	Assessment of methodological quality
To vaccinate or not to vaccinate!? Predictors of willingness to receive Covid-19 vaccination in Europe, the US, and China [30]	Júlia Brailovskaia,Sílvia Schneider,Jürgen Margraf20) / Plos One / 2021	Vaccination willingness and its potential predictors in representative online samples across nine countries (China, France, Germany, Poland, Russia, Spain, Sweden, UK, USA)	9264 Nine countries (China, France, Germany, Poland, Russia, Spain, Sweden, UK, USA)	Cross-sectional Predominantly female sample, aged 55 or over, with partners, with the working and middle classes being the most frequent	Russia had the lowest rate of vaccinated participants (18%). The rate of participants who wanted to be vaccinated but had not yet been vaccinated was highest in Spain (54.6%), with the lowest rate being in the US (8.7%). Social media use was a significant negative predictor in three countries (PL, SV, US). Stress symptoms served as a significant negative predictor in Germany and the US	5/8
Examining confidence and hesitancy towards COVID-19 vaccines: A cross-sectional survey using in-person data collection in rural Ghana [23]	OK Afreh, P. Angwaawie, Ejk Attivor, LA Boateng, K Brackstone, Chefe de MG, AK Manyeh, Gaa Vidzro/ National library of medicine/2022	To assess vaccine hesitancy and confidence in Nkwanta South, a rural municipality in the Oti region, Ghana	1500 Ghana	Cross-sectional Of the total number of interviewees, 53.1% were men, the average age was 40 years, 32.1% had no formal education, 70.0% were married and 80.7% were employed. By religion, 74.7% identified themselves as Christians	53.3% reported not being vaccinated against COVID-19. 69.4% reported willingness to get the vaccine when available, 23.7% said they would not be willing to get vaccinated, 6.9% said they were unsure. Overall, this represented 30.6% hesitancy within the group. Key predictors of hesitancy among our participants included high levels of vaccine distrust, concern about side effects, lack of information, being female, more years of education, and being Christian	6/8

Predictors of COVID-19 Vaccine Hesitancy: Socio-Demographics, Co-Morbidity, and Past Experience of Racial Discrimination [33]	Elena Savoia, Rachael Piltch-Loeb,Beth Goldberg, Cynthia Miller-Idriss,Brian Hughes,Alberto Montrond,Juliette Kayyem, Marcia A./ Vaccine/2021	To explore predictors of COVID-19 vaccine hesitancy, including sociodemographic factors, comorbidity, risk perception, and experience of discrimination, in a sample of the US population	2.650 USA	Cross-sectional The majority of respondents were between the ages of 25 and 44 (66%), were male (53%), and worked in the healthcare sector (61%) 66% were white and non-Hispanic	The factors associated with vaccine hesitancy were racial discrimination and confidence in the safety and efficacy of vaccines. The results of the study emphasize the need to enhance public opinion monitoring strategies by gathering information on concerns and reasons for hesitancy regarding the COVID-19 vaccine that get to the roots of this hesitancy, in addition to the use of responses related to safety and efficacy	7/8
Factors associated with the unwillingness of Jordanians, Palestinians and Syrians to be vaccinated against COVID-19 [7]	Sima Zein,Sarah B. Abdallah,Ahmed Al-Smadi,Omar Gammoh,Wajdy J. Al-Awaida,Hanan J. Al-Zein/PLOS Neglected Tropical Diseases/2021	To assess the intention to be vaccinated against COVID-19 and identify predictors and reasons among participants who are unwilling/hesitant to be vaccinated	8.619 3 Middle Eastern countries (Jordan, West Bank and Syria)	Cross-sectional 69.4% were women, approximately half (48.2%) were between 18 and 35 years old, more than half of the participants had a bachelor's degree (51.7%), did not work in a health-related field (77.6%), were employed (61.1%) and were married (60.1%)	32.2% of participants intended to be vaccinated, these participants willing to be vaccinated were between 18 and 35 years old and participants had a high school diploma or less. Factors associated with hesitancy were the lack of rigorous evaluation of the vaccine by the FDA and the possible long-term health risks associated with vaccines	5/8
Factors for hesitancy towards vaccination against COVID-19 among the adult population in Puducherry, India - a cross sectional study [32]	Raja Jeyapal Dinesh, Rajendran Dhanalakshmi, Priskilla Johnson Jency, Adinarayanan Srividya, Balakrishnan Vijayakumar, Ashwani Kumar/ BMC Public Heath/2023	To estimate the magnitude of COVID-19 vaccination hesitancy and determine its associated factors in the community	776 India	Cross-sectional The average age of participants was 43.3 years, and the majority were female (67.0%) 34% lived in urban areas, 64.3% had completed high	More than half of the participants were hesitant about vaccination. Fear of needles, fear of adverse effects due to smoking cigarettes and/or drinking alcohol, belief in naturally acquired immunity, being busy with other work, and religious reasons were the predictors of hesitancy	6/8

				school, and 61.1% were unemployed		
COVID-19 vaccine hesitancy trends in Ghana: a cross- sectional study exploring the roles of political allegiance, misinformation beliefs, and sociodemographic factors [24]	Ken Brackstone, Kirchuffs Atengble, Michael Head, Laud Boateng/Pan Afr Med J/2022	To detect levels of COVID-19 vaccine hesitancy among unvaccinated individuals in Ghana and observe their trends over time, and to identify independent predictors associated with vaccine hesitancy among unvaccinated individuals	1.067 Ghana	Cross-sectional 62.3% had completed higher education. Over 60% of participants reported being single (62.0%), 58.7% reported living in an urban area, 45.7% reported being unemployed, and 82.5% reported being Christian	Hesitancy decreased between August 2020 and March 2021. However, hesitancy increased in June 2021 and continued to increase further in February 2022. The main reasons for refusing the vaccine included not having enough information about the vaccine and concerns about vaccine safety. Among the main groups most likely to express hesitancy were Christians, urban residents, opposition party voters, women, individuals with higher education, individuals who received information about COVID-19 from internet sources, and individuals who expressed uncertainty about their beliefs about COVID-19 misinformation	7/8
Acceptance of a COVID-19 Vaccine in Nigeria: A Population-Based Cross-Sectional Study [13]	Ekaete Alice Tobin, Martha Okonofua e Azuka Azeke/Cureus/2021	To assess the willingness to accept a future COVID-19 vaccine in Nigeria and associated factors	1228 Nigeria	Cross-sectional 53% were women. The average age of respondents was 32 years; About 75% of participants had at least a college education	50.8% of respondents were willing to accept a COVID-19 vaccine when it became available in the country, and these individuals belonged to the risk group, were elderly and were male. Factors associated with hesitation were not trusting the government, being Christian, being self-employed and having concerns about safety and efficacy	5/8
Sociodemographic predictors of and main reasons for COVID-19 vaccine hesitancy in eastern Oslo: a cross-sectional study [29]	Lara Steinmetz/BMC Public Health/2022	To identify the sociodemographic predictors of vaccine hesitancy, the main reasons for vaccine hesitancy, and how these reasons are	5442 Oslo	Cross-sectional Almost 60% were women, with ages (30–44, 45–59, 60+) constituting approximately 30% each. 78.6% of	Of those who had been offered the vaccine (77.1% of participants), 87.1% responded that they were not interested and 11.9% were unsure. It was observed that those over 45 years of age had significantly lower odds ratios compared to those aged 18 to	6/8

		explained by sociodemographic characteristics during the COVID-19 pandemic		participants completed a university degree	29 years, i.e. less hesitancy. Additionally, individuals with more than 4 years of college education were almost 50% less likely to be hesitant compared to those who had completed primary school. Participants in the highest income group were also less likely to be hesitant compared to the lowest income group	
The impact of information sources on COVID-19 vaccine hesitancy and resistance in sub-Saharan Africa [25]	Uchechukwu L. Osuagwu, Khathutshelo P. Mashige, Godwin Ovenseri-Ogbomo, Esther Awazzi Envuladu, Emmanuel Kwasi Abu, Chundung Asabe Miner, Chikasirimobi G. Timothy, Bernadine N. Ekpenyong, Raymond Langsi, Onyekachukwu M. Amiebenomo, Richard Oloruntoba13 , Piwuna Christopher Goson14, Deborah Donald Charwe, Tanko Ishaya e Kingsley E. Agho/ BMC Public Heath/2023	Examining the impact of information sources on COVID-19 vaccine hesitancy and resistance in sub-Saharan Africa	2572<br><br>Africa	Cross-sectional<br>54% were men, 80% of the participants had higher education, about a third were between 18 and 28 years old (36.1%) and more than half of them were not married (56.0%). About 80% of the participants were employed	Television and Facebook were the main sources of up-to-date information for participants during the pandemic. However, those who obtained information from social media platforms were found to be twice as likely to resist COVID-19 vaccines compared to non-users. Those who relied on television, healthcare professionals, friends, and family for up-to-date information were more likely to resist the vaccine than their counterparts. In contrast, the odds of resisting the vaccine were significantly reduced among those who reported that the newspaper was their main source of information during the pandemic	6/8
Characteristics associated with COVID-19 vaccine hesitancy [16]	Liyousew G. Borga, Andrew E. Clark, Conchita D'Ambrosio e Anthony Lepinteur./BMC Scientific Reports/ 2022	Understanding the characteristics associated with COVID-19 vaccine hesitancy	4.899<br>Brazil	Cross-sectional<br>Majority, middle-aged, female, white, married, of lower socioeconomic status and with completed higher education	According to this study, greater vaccine hesitancy was associated with low education, low family income, younger population and low trust in government conduct. Regarding gender, greater hesitancy was observed in the female population, but the authors believe that there is no direct association between being a woman and greater hesitancy, since	8/8

					the men in this study were generally older and the older population showed greater adherence to vaccination. 59% of the participants were vaccinated	
Relationship between knowledge, attitudes, and practices and COVID-19 vaccine hesitancy: A cross-sectional study in Taizhou, China [14]	Xiao-Qing Lin; Mei-Xian Zhang; Yan Chen; Ji-Ji Xue; He-Dan Chen; Tao-Hsin Tung; and Jian-Sheng Zhu./Front Med (Lausanne) .2022.	To explore COVID-19 vaccine hesitancy in Chinese adults and analyze the relationship between knowledge, attitudes, practices (KAP) and COVID-19 vaccine hesitancy	1.788 China	Cross-sectional 74.9% were female, the average age of respondents was 41 years, 58.7% of the people lived in urban areas, 47.7% had an education level of Junior College and above, while 29.3% had an education level of Junior Secondary and below	45.2% of people were hesitant about getting vaccinated against COVID-19. Low perceptions of COVID-19 vaccine safety and efficacy in adults are the most important risk factor for COVID-19 vaccine hesitancy. People who know more about COVID-19 vaccination are less hesitant. People who have not independently sought information about the COVID-19 vaccine are more likely to be skeptical	8/8
Determinants of COVID-19 vaccine acceptance and hesitancy: a cross-sectional study in Saudi Arabia [20]	Amar Ibrahim Omer Yahia um,b, Abdullah Mohammed Alshahranic, Wael Gabir H. Alsulmie,Mohammed Mesfer M. Alqarnie, Tamim Khalid Abdullah Abdulrahime, Waleed Faya H Hebae, Turco Ayidh A. Alqarnie,Khalid Ali Z Alharthie, e Abdullah Ali A. Buhran./Hum Vaccin Immunother/2021	To determine COVID-19 vaccine acceptance and hesitancy rates and investigate factors influencing vaccine acceptance and hesitancy	531 Saudi Arabia	Cross-sectional The majority of respondents were male (59.7%), Saudi (98.3%), urban residents (72.9%), aged 34–49 (40.9%), had completed higher education or postgraduate studies (81.8%), were employed (73.5%), were married (64.4%), and came from middle-income families (48.4%)	61.8% were willing to take the COVID-19 vaccine, while 38.2% were not. COVID-19 vaccine hesitancy was higher among women (44.9%), those aged 34-49 years (47.9%), those who were married (41.9%), employed (39.7%), had lower educational attainment (40%), and urban residents (40.8%). The main reason for COVID-19 vaccine acceptance was to protect themselves and others, while concerns about vaccine safety were the main reason for vaccine hesitancy	7/8

COVID-19 vaccine hesitancy in Egypt: a cross-sectional study [15]	May Ahmed Shawki; Ahmed Kamel; Shaza Gamal; Maggie Magdy Abbassi; Samar Farghali Farid; Nirmeen Ahmed Sabry./J Infect Dev Ctries/2023. .	To determine the factors associated with COVID-19 vaccine acceptance.	24.376 Egypt	Cross-sectional Women represented more than two-thirds of the study sample (70.5%) and 18-24 years was the most commonly reported age range. About one-third of the sample were healthcare professionals	53% of participants received or registered to receive the vaccine, while 47% refused to be vaccinated. Regression analysis revealed that male gender, secondary education, older age, married or divorced status, presence of comorbidities, and higher level of vaccine knowledge were significantly associated with high vaccine acceptance. The most important vaccine attributes that influenced vaccine selection in the current study were efficacy and safety	8/8
Individual determinants of COVID-19 vaccine hesitancy [9]	Philip Gerretsen, Julia Kim, Fernando Caravaggio, Lena Quilty, Marcos Sanches, Samantha Wells, Eric E Brown, Branka Agic, Bruce G Pollock, Ariel Graff-Guerrero/Plos One/2021.	Identify the determinants of COVID-19 vaccine hesitancy based on the World Health Organization's '3Cs' model (i.e., confidence, complacency, and convenience) in the United States (US) and Canada	7.678 USA	Cross-sectional The mean age was 47 years, and 50.8% were women. The majority of participants identified their race as white (68.3%). A large proportion of our sample identified with a religion (67.5%), with the largest representation being Christian (45%)	Vaccination distrust and lower perceived severity of COVID-19 were the main determinants of vaccine hesitancy. Right-wing political affiliation, higher risk propensity, and fewer negative mental health effects of the COVID-19 pandemic were the main sociodemographic and psychological determinants. Other sociodemographic determinants included younger age, females, race, and employment status. Vaccine distrust and complacency explained 38% and 21% of the variance in vaccine hesitancy, respectively; while sociodemographic and psychological determinants explained 13% and 11% of the variance in vaccine hesitancy, respectively	8/8
Attitudes toward the sars-cov-2 vaccine: Results from the saudi residents' intention to get vaccinated against covid-19 (srigvac) study [26]	Sami H. Alzahrani, Mukhtiar Baig , Mohammed W. Alrabia , Mohammed R. Algethami , Meshari M. Alhamdan , Nabil A. Alhakamy , Hani Z. Asfour , Tauseef Ahmad/Vaccine/2021	To assess the Saudi public's intention to get vaccinated against COVID-19 and explore the demographic determinants associated	3.048 Saudi Arabia	Cross-sectional The majority of respondents were between 18 and 44 years old (85.1%) and were Saudi	52.9% intend to get vaccinated, 26.8% were unsure and 20.3% refused vaccination. Vaccine hesitancy was significantly higher among women. The likelihood was lower among Saudis, those with less than a high	5/8

		with their intentions, as well as the reasons for vaccine hesitancy		nationals (89.5%). More than half of the population were women (60.1%) residing in the Western Region (57.0%) and were currently employed (52.9%)	school education, those with perceived risks of COVID-19 and residents of the southern region	
Covid-19 vaccine acceptance in the democratic republic of congo: A cross-sectional survey [34]	John D Ditekemena, Dalau M Nkamba, Armand Mutwadi, Hypolite M Mavoko, Joseph Nelson Siewe Fodjo, Christophe Luhata, Michael Obimpeh, Stijn Van Hees, Jean B Nachega, Robert Colebunders/ Vaccine/2021.	To investigate the level of willingness to be vaccinated against COVID-19 in the Democratic Republic of the Congo	4.131 Democratic Republic of the Congo	Cross-sectional The average age was 35 years; 68.4% were women and 71% had primary or secondary education.	Factors associated with vaccine hesitancy were racial discrimination, trust in the safety and efficacy of vaccines, and false beliefs about the vaccine. 44.1% had no intention of getting vaccinated	6/8
Interaction between age and health conditions in the intention to be vaccinated against COVID-19 in Thailand [18]	Sakun Boon-Itt, Nopadol Rompho, Sompong Jiarnkamolchurn, Yukolpat Skunkan /Hum Vaccin Immunother /2021.	To study the sociodemographic factors as well as the interaction between age groups and health conditions in relation to the intention to be vaccinated against coronavirus disease 2019 (COVID-19) in Thailand.	862 Thailand	Cross-sectional The majority of respondents were women, representing 63.9%. 61.7% of respondents were between 18 and 44 years old, 45.3% had higher education and 57.7% were single	55.6% would likely receive a COVID-19 vaccine. Men, those aged 18 to 44, those with comorbidities, and those with higher income were more likely to get vaccinated	5/8
Vaccine Hesitancy and Exposure to Misinformation: a Survey Analysis [8]	Stephen R. Neely, Christina Eldredge, Robin Ersing, and Christa Remington/J Gen Intern Med /2022.	To better understand common objections to COVID-19 vaccination and the potential effects of misinformation on vaccine decisions	600 USA	Cross-sectional 52% of the sample was female, aged between 45-64 years (32%), 77.3% were white	Factors associated with adherence: Democratic Party politics, white race, male gender, college degree, and age 65 or older. Factors associated with hesitancy: concern about potential side effects of the vaccine; fears that the vaccines were developed too quickly to be adequately tested; self-identified Republicans. 63.4% of participants were vaccinated	5/8

COVID-19 Vaccination Willingness and Associated Factors in Japanese Primary Care Patients: A Cross-Sectional Study [10]	Natsuki Kajikawa 1 2, Shoji Yokoya 1 2, Tetsuhiro Maeno 3/J Prim Care Community Health /2022.	To investigate perceptions and attitudes towards COVID-19 and its vaccination, as well as willingness to undergo COVID-19 vaccination and associated factors, immediately prior to the start of COVID-19 vaccination	717 Japan	Cross-sectional The median age was 67 years, 53.7% were considered elderly and 54.8% were women. 86.6% visited doctors regularly. Influenza vaccination coverage in the 2020/21 season was 71.1%	Willingness to be vaccinated was associated with older age, male gender, influenza vaccination in the 2020/2021 season, high perceived susceptibility, belief in vaccine efficacy, high perceived susceptibility to vaccine-related adverse events, and high adherence to social norms. 70% of participants were vaccinated	5/8
COVID-19 Vaccine Hesitancy in Italy: Predictors of Acceptance, Fence Sitting and Refusal of the COVID-19 Vaccination [11]	Cristina Zarbo 1, Valentina Candini 1, Clarissa Ferrari 2, Miriam d'Addazio 1, Gemma Calamandrei 3, Fabrizio Starace 4, Marta Caserotti 5, Teresa Gavaruzzi 5, Lorella Loteria 5, Alessandra Tasso 6, Manuel Zamparini 1, Giovanni de Girolamo 1/ Frontiers/2022.	To assess sociodemographic, psychological and behavioral factors, as well as attitudes and beliefs that influence COVID-19 vaccination hesitancy in the general population of Italy.	2015 Italy	Cross-sectional Average age 43-50 years, majority male, employed, with average education of 0-8 years	Sociodemographic characteristics (such as age, education, economic status, having a chronic illness, experience with COVID-19), psychological well-being, attitudes and beliefs (such as trust in media sources and institutions, trust in institutions, agreement with restrictions, perceived risk of COVID-19, conspiracy mentality) and behaviors (i.e., protective behavior against the virus, frequency of use of media or institutional information sources)	6/8
Deliberation, Dissent, and Distrust: Understanding Distinct Drivers of Coronavirus Disease 2019 Vaccine Hesitancy in the United States [27]	Bonde Khai Hoan, Sahar Saeed , Cory Bradley , Raposa Branson , Ingrid Eshun-Wilson , Aaloke Mody ,e Elvin Geng/Clin Infect Dis /2022	To assess predictors of vaccine hesitancy and vaccine rejection	459.235 USA	Cross-sectional The average age was 55 years, 59.8% were women, white (76%), 39.2% had high school education or less and 55.3% were married	Females, younger age groups, black Americans, multiracial people, those with less education, lower income, and those living in Republican-leaning states were hesitant to take the COVID-19 vaccine. 24.6% of participants were vaccinated	6/8
Willingness and Perceptions Regarding COVID-19 Vaccine Booster	J Cunningham-Erves 1, O Jorge 2, M Sanderson 3, E Stewart 1, SW Jin 4, J. Davis 5, Sua Majestade Brandt 6/Front	To assess willingness to receive booster dose of COVID-19 vaccine and	787 Pakistan	Cross-sectional 69.6% were women, 75.3% were in the	A 77.8% willingness to receive a booster dose was reported. Participants showed no or low levels	5/8

Dose in Pakistani Vaccinated Population: A Cross-Sectional Survey [19]	Public Health ./2022.	identify predictors and factors of willingness and hesitancy in vaccinated population of Pakistan.		age group of 18 to 30 years old, 53.5% were university students or had a bachelor's degree	of fear associated with a booster dose (47.3%). 60.1% agreed that it was safe to receive an additional dose of the vaccine, with 44.1% agreeing that boosters are effective against coronavirus variants. Independent predictors of willingness included the absence of comorbidities, while being unwilling to pay for the booster dose was a predictor of hesitancy	
A National Survey Assessing COVID-19 Vaccine Hesitancy Among Arab Americans [28]	Siwaar Abouhala 1, Alzahra Hamidaddin 2, Mahdi Taye 3, Delaney J Glass 4, Noor Zanial 5, Feda Hammood 3, Farah Allouch 6, Nadia N Abuelezam 7J Racial Ethn Health Disparities/2022.	To analyze predictors of vaccine intention among Arab Americans.	638 USA	Cross-sectional 52% were women, most were between the ages of 18 and 25. 30.4% reported an annual household income of more than $100,000, and 25.1% reported an income of less than $5,000.	More than half (56.7%) of respondents reported an intention to get vaccinated with a COVID-19 vaccine, 35.7% reported uncertainty, and 7.5% reported being unlikely to get vaccinated. Of those unlikely to get vaccinated, 72.9% were women and 85.4% reported moderate to high religiosity. Arab American women were more likely to be uncertain about their vaccine intention or unlikely to get vaccinated than men in this sample	5/8
COVID-19 vaccine hesitancy among adults in Hawassa City Administration, Sidama Region, Ethiopia: A community-based study [31]	Seblewongel Yohannes 1, Akalewold Alemayehu 2, Yohannes Markos Woldesenbet 3, Temesgen Tadele 1, Desta Dangiso 1, Muntasha Birhanu 1, Endrias Markos Woldesemayat 2/Front Public Health ./2023	To assess COVID-19 vaccine hesitancy and associated factors among adults in Hawassa City, southern Ethiopia	622 Ethiopia	Cross-sectional 53.1% were between 18 and 29 years old and 51.4% were male. Employed participants constituted 60.9% and 46.8% participants had education above high school. The majority of participants were	64.3% had a high level of knowledge about the COVID-19 vaccine, 68.3% had a positive attitude towards the COVID-19 vaccine. The level of vaccine hesitancy was 26.5% and vaccine acceptance was 73.5%. The main reason for willingness to take the vaccine was to protect oneself from COVID-19 and for unwillingness, it was fear of the vaccine. Mass media was the main source of information about the vaccine (75.9%). Age, religion, history of COVID-19 disease, knowledge related to COVID-19 vaccine and	6/8

				married, 63.5% and Protestant 64.6%	attitude towards COVID-19 vaccine were factors associated with vaccine hesitancy	
COVID-19 vaccine hesitancy in six geopolitical zones in Nigeria: a cross-sectional survey [17]	Babatunde Oluwatosin Ogunbosi 1, Michael Abel Alao 2, Olayinka Rasheed Ibrahim 3, Adaeze Chikaodinaka Ayuk 4, Rasheedat Mobolaji Ibraheem 5, Chioma Laura Odimegwu 4, David Chibuike Ikwuka 6, Patricia Akintan 7, OreOluwa Morakinyo 2, Ayomide Toluwanimi Adeyemi 2, Ridwan Muhammad Jega 8, Temitayo Folorunso Olowookere 9, Olaseinde Emmanuel Bello 10, Bilkis Iyabo Owolabi 11, Abejegah Chukwuyem 12, Lawan Maryah Bukar 13, Aliu Rasaki 14, Amudalat Issa 15, Atana Uket Ewa 16, Regina Oladokun 1, Olusegun Olusina Akinyinka/ Pan Afr Med J/2022.	To understand the prevalence and factors associated with COVID-19 vaccine hesitancy across the six geopolitical zones of Nigeria.	1615 Nigeria	Cross-sectional The average age was 36 years, 52.4% were men. More than half were health professionals (58.4%), 97.4% had at least a high school education and the majority, 60.5%, belonged to the upper social class	The prevalence of hesitancy was 68.5%, residence in the Northeast and Northwest geopolitical zones, the Igbo ethnic group, Christians, nurses, pharmacists, and participants with no confidence in foreign vaccines were more likely to have VH	7/8
COVID-19 Vaccine Hesitancy among Population in Jazan Region of Saudi Arabia [35]	Manal Almalki 1,Mohammed Kotb Sultan 2,3,Mohammed Abbas 3,Ajiad Alhazmi 3ORCID,Yasser Hassan 3ORCID andJoe Varghese/Healthcare/2023	To investigate the extent of vaccine hesitancy related to the COVID-19 vaccine and associated factors in the Jazan region.	569 Saudi Arabia	Cross-sectional There were more men than women, and the majority were Saudi citizens (96.7%). More than half of the participants were between 20 and 29 years old	(36.9%) were vaccine hesitant. Concern about adverse side effects after vaccination was the most reported reason for vaccine hesitancy (42.6%), followed by beliefs that the vaccine was unsafe or ineffective (15.5%). Data analysis revealed that people living in cities in Jazan province or those who did not have a family history of COVID-19 infection were more likely to be vaccine hesitant	6/8
Sociodemographic and Behavioral Predictors of COVID-19 Vaccine Hesitancy in Pakistan [36]	Osama Al-Wutayd 1, Rehana Khalil 1, Allah Bachayo Rajar 2/J Multidiscip Healthc . 2021	To determine the proportion and predictors of COVID-19 vaccine hesitancy among adults in Pakistan.	1014 Pakistan	Cross-sectional 44% were under 30 years of age. 53% were male, 67% of the respondents resided in urban	35.8% were hesitant to receive the COVID-19 vaccine. Reasons for hesitancy included concerns about side effects (42.4%), belief in conspiracy theories (20.1%), perceived vaccine ineffectiveness	8/8

				areas of the country. Regarding educational level, 53% of the study respondents had a college degree, and 46% reported that they were employed. 67% reported a monthly household income between 131 USD to 2,083 USD	(13.2%), and perceived protection through precautionary measures (12.6%). Urban residence (AOR 2.34, 95% CI 1.54–3.57), reservations about vaccine safety (AOR 3.29, 95% CI 1.68–6.44), uncertainty about vaccine efficacy (AOR 2.70, 95% CI 1.50–4.86), failure of the vaccine to reduce hospitalization and death (AOR 6.36, 95% CI 4.01–10.22), and lack of felt need for vaccination awareness among the public.	
Social Environmental Predictors of COVID-19 Vaccine Hesitancy in India: A Population-Based Survey [21]	Srikanth Umakanthan 1, Maryann M Bukelo 2, Mario J Bukelo 3, Sonal Patil 4, Naveen Subramaniam 4, Ria Sharma 5/Vaccines/2022	To assess the intention to get vaccinated	2000 India	Cross-sectional 50.69% were women, aged between 31-40 years, 60.41% with higher education	Demographic factors were not influential on vaccination intention. Both sources (official and unofficial sources) and trust (trust in the social environment and in vaccines) are significantly and positively correlated with vaccination intention. Receiving information from official and unofficial sources were significant positive predictors of trust in the social environment, but were not significant predictors of trust in vaccines	8/8
Determinants of COVID-19 Vaccine Acceptance Among the General Adult Population in Saudi Arabia Based on the Health Belief Model: A Web-Based Cross-Sectional Study [22]	Ezzuddin A Okmi 1, Emad Almohammadi 1, Olfat Alaamri 1, Rasha Alfawaz 2, Naif Alomari 3, Marwah Abdulaziz Saleh Alnughaymishi 2, Sulaiman Alsuwailem 1, Naseem J Moafa/Cureus . 2022	To assess demographic characteristics and sociopsychological variables that affect willingness to receive the COVID-19 vaccine among the general adult population	1939 Saudi Arabia	Cross-sectional 57.2% were men. The average age was 39 years, 50.7% had graduated from a university. The majority (79.5%) were married, and about 30% of the participants had a monthly income of less than 5000 SR.	Men were 1.29 times more likely to receive the COVID-19 vaccine than women, and those who were or had been healthcare workers (HCWs) were 1.43 times more likely to receive the COVID-19 vaccine compared to those who had never been a HCW. We found that perceiving the risk of contracting COVID-19 and perceiving the severity of the disease were positively associated with willingness to receive the vaccine. Perceived	7/8

				Half (50.4%) of the participants were government employees, and more than a third (38.8%) worked or had worked as health professionals	barriers, such as vaccine ineffectiveness or believing that the vaccine is just a media hype, were negative predictors of vaccine acceptance. Additionally, perceiving benefits, such as life returning to normal, and recognizing the importance of the annual flu vaccine were found to be positive predictors of vaccine acceptance. Finally, we also found that action cues were positively associated with vaccine acceptance, i.e., participants who were encouraged by their doctors and family or friends were more willing to receive the COVID-19 vaccine than those who were not	
COVID-19 Vaccine Hesitancy and Its Associated Factors in Japan [12]	Ryo Okubo 1, Takashi Yoshioka 2, Satoko Ohfuji 3, Takahiro Matsuo 4, Takahiro Tabuchi/Vaccines/2021.	To examine the proportion of COVID-19 vaccine hesitancy in the Japanese population	23.142 Japan	Cross-sectional 49.2% were women and 25.5% were between 65 and 79 years old, 46.2% with income between 1 to 6 (million JPY/year), married people accounted for 65.9%, and 42.1% had high school education.	The most common reason cited for not getting vaccinated was concerns about adverse reactions in over 70% of respondents. Factors associated with hesitancy were female gender, living alone, low socioeconomic status, and presence of severe psychological distress, especially among older respondents	8/8

Supplementary Material Table S2. - Preparation of the study question according to the PEO strategy, Ribeirão Preto, 2024.

Acronym	Definition	Descriptors
**P**	Patient or Problem	General population
**E**	Exposure	Associated factors
**O**	*Outcome*	Adherence or hesitancy to the COVID-19 vaccine

Supplementary Material Table S3. - Bibliographic search strategy used in the systematic review on the factors associated with vaccine adherence or hesitancy against COVID-19, according to the databases researched, Ribeirão Preto, 2024.

Database	Search Strategy
Embase®	#6 #5 AND [embase]/lim<br>#5 #1 AND #2 AND #3 AND #4<br>#4 ('risk factors'/exp OR 'risk factors' OR 'associated factors' OR 'predictive factors' OR 'predictors'/exp OR predictors) AND [2020-2024]/py<br>#3 ('refusal'/exp OR refusal OR hesitancy OR 'acceptability'/exp OR acceptability OR 'acceptance'/exp OR acceptance OR 'adherence'/exp OR adherence) AND [2020-2024]/py<br>#2 (('covid19'/exp OR covid19 OR 'covid 19'/exp OR 'covid 19' OR sarscov2 OR 'sars cov2' OR 'sars cov 2'/exp OR 'sars cov 2' OR 'sars'/exp OR sars) AND cov AND ('2'/exp OR 2) OR 'sars cov2' OR 'coronavirus'/exp OR coronavirus) AND [2020-2024]/py<br>#1 ('vaccine'/exp OR vaccine OR 'vaccination'/exp OR vaccination OR 'immunization'/exp OR immunization) AND [2020-2024]/py
Scopus	TITLE-ABS-KEY(Vaccine OR vaccination OR immunization) AND TITLE-ABS-KEY(Covid19 OR Covid-19 OR "covid 19" OR sarscov2 OR sars-cov2 OR sars-cov-2 OR sars cov 2 OR sars-cov2 OR coronavirus) AND TITLE-ABS-KEY(Refusal OR Hesitancy OR acceptability OR acceptance OR adherence) AND TITLE-ABS-KEY("Risk factors" OR "associated factors" OR "predictive factors" OR predictors)
PUBMED	(("vaccin"[Supplementary Concept] OR "vaccin"[All Fields] OR "vaccination"[MeSH Terms] OR "vaccination"[All Fields] OR "vaccinable"[All Fields] OR "vaccinal"[All Fields] OR "vaccinate"[All Fields] OR "vaccinated"[All Fields] OR "vaccinates"[All Fields] OR "vaccinating"[All Fields] OR "vaccinations"[All Fields] OR "vaccination s"[All Fields] OR "vaccinator"[All Fields] OR "vaccinators"[All Fields] OR "vaccine s"[All Fields] OR "vaccined"[All Fields] OR "vaccines"[MeSH Terms] OR "vaccines"[All Fields] OR "vaccine"[All Fields] OR "vaccins"[All Fields] OR ("vaccin"[Supplementary Concept] OR "vaccin"[All Fields] OR "vaccination"[MeSH Terms] OR "vaccination"[All Fields] OR "vaccinable"[All Fields] OR "vaccinal"[All Fields] OR "vaccinate"[All Fields] OR "vaccinated"[All Fields] OR "vaccinates"[All Fields] OR "vaccinating"[All Fields] OR "vaccinations"[All Fields] OR "vaccination s"[All Fields] OR "vaccinator"[All Fields] OR "vaccinators"[All Fields] OR "vaccine s"[All Fields] OR "vaccined"[All Fields] OR "vaccines"[MeSH Terms] OR "vaccines"[All Fields] OR "vaccine"[All Fields] OR "vaccins"[All Fields]) OR ("immune"[All Fields] OR "immuned"[All Fields] OR "immunes"[All Fields] OR "immunisation"[All Fields] OR "vaccination"[MeSH Terms] OR "vaccination"[All Fields] OR "immunization"[All Fields] OR "immunization"[MeSH Terms] OR "immunisations"[All Fields] OR "immunizations"[All Fields] OR "immunise"[All Fields] OR "immunised"[All Fields] OR "immuniser"[All Fields] OR "immunisers"[All Fields] OR "immunising"[All Fields] OR "immunities"[All Fields] OR "immunity"[MeSH Terms] OR "immunity"[All Fields] OR "immunization s"[All Fields] OR "immunize"[All Fields] OR "immunized"[All Fields] OR "immunizer"[All Fields] OR "immunizers"[All Fields] OR "immunizes"[All Fields] OR "immunizing"[All

	Fields])) AND ("covid 19"[MeSH Terms] OR "covid 19"[All Fields] OR "covid19"[All Fields] OR ("covid 19"[All Fields] OR "covid 19"[MeSH Terms] OR "covid 19 vaccines"[All Fields] OR "covid 19 vaccines"[MeSH Terms] OR "covid 19 serotherapy"[All Fields] OR "covid 19 nucleic acid testing"[All Fields] OR "covid 19 nucleic acid testing"[MeSH Terms] OR "covid 19 serological testing"[All Fields] OR "covid 19 serological testing"[MeSH Terms] OR "covid 19 testing"[All Fields] OR "covid 19 testing"[MeSH Terms] OR "sars cov 2"[All Fields] OR "sars cov 2"[MeSH Terms] OR "severe acute respiratory syndrome coronavirus 2"[All Fields] OR "ncov"[All Fields] OR "2019 ncov"[All Fields] OR (("coronavirus"[MeSH Terms] OR "coronavirus"[All Fields] OR "cov"[All Fields]) AND 2019/11/01:3000/12/31[Date - Publication])) OR "covid 19"[All Fields] OR "sarscov2"[All Fields] OR "sars-cov2"[All Fields] OR ("sars cov 2"[MeSH Terms] OR "sars cov 2"[All Fields] OR "sars cov 2"[All Fields]) OR ("sars cov 2"[MeSH Terms] OR "sars cov 2"[All Fields] OR "sars cov 2"[All Fields]) OR "sars-cov2"[All Fields] OR ("coronavirus"[MeSH Terms] OR "coronavirus"[All Fields] OR "coronaviruses"[All Fields])) AND ("garbage"[MeSH Terms] OR "garbage"[All Fields] OR "refuse"[All Fields] OR "refuses"[All Fields] OR "refusal"[All Fields] OR "refusals"[All Fields] OR "refused"[All Fields] OR "refuser"[All Fields] OR "refusers"[All Fields] OR "refusing"[All Fields] OR ("hesitance"[All Fields] OR "hesitancies"[All Fields] OR "hesitancy"[All Fields] OR "hesitant"[All Fields] OR "hesitate"[All Fields] OR "hesitated"[All Fields] OR "hesitating"[All Fields] OR "hesitation"[All Fields] OR "hesitations"[All Fields]) OR ("accept"[All Fields] OR "acceptabilities"[All Fields] OR "acceptability"[All Fields] OR "acceptable"[All Fields] OR "acceptably"[All Fields] OR "acceptance"[All Fields] OR "acceptances"[All Fields] OR "acceptation"[All Fields] OR "accepted"[All Fields] OR "accepter"[All Fields] OR "accepters"[All Fields] OR "accepting"[All Fields] OR "accepts"[All Fields]) OR ("accept"[All Fields] OR "acceptabilities"[All Fields] OR "acceptability"[All Fields] OR "acceptable"[All Fields] OR "acceptably"[All Fields] OR "acceptance"[All Fields] OR "acceptances"[All Fields] OR "acceptation"[All Fields] OR "accepted"[All Fields] OR "accepter"[All Fields] OR "accepters"[All Fields] OR "accepting"[All Fields] OR "accepts"[All Fields]) OR ("adherance"[All Fields] OR "adhere"[All Fields] OR "adhered"[All Fields] OR "adherence"[All Fields] OR "adherences"[All Fields] OR "adherent"[All Fields] OR "adherents"[All Fields] OR "adherer"[All Fields] OR "adherers"[All Fields] OR "adheres"[All Fields] OR "adhering"[All Fields])) AND ("Risk factors"[All Fields] OR "associated factors"[All Fields] OR "predictive factors"[All Fields] OR ("predictor"[All Fields] OR "predictors"[All Fields]))) AND (2020:2024[pdat])
LILACS*	(vaccine OR vaccination OR immunization OR vacina OR vacinação OR imunização OR vacuna OR vacunación OR inmunización) AND (covid19 OR covid-19 OR "covid 19" OR sarscov2 OR sars-cov2 OR sars-cov-2 OR sars cov 2 OR sars-cov2 OR coronavirus) AND (refusal OR hesitancy OR acceptability OR acceptance OR adherence OR recusa OR hesitação OR aceitabilidade OR aceitação OR adesão OR rechazo OR vacilación OR aceptabilidad OR aceptación OR adhesión) AND ("Risk factors" OR "associated factors" OR "predictive factors" OR predictors OR "Fatores de risco" OR "fatores associados" OR "fatores preditivos" OR preditores OR "Factores de riesgo" OR "factores asociados" OR "factores predictivos" OR predictores) AND (db:("LILACS"))
Web of Science	Vaccine OR vaccination OR immunization (Topic) and Covid19 OR Covid-19 OR "covid 19" OR sarscov2 OR sars-cov2 OR sars-cov-2 OR sars cov 2 OR sars-cov2 OR coronavirus (Topic) and Refusal OR Hesitancy OR acceptability OR acceptance OR adherence (Topic) and "Risk factors" OR "associated factors" OR "predictive factors" OR predictors (Topic)
